# Isolation, Structures, and Bioactivities of Polysaccharides from *Achyranthes bidentata*: A Review

**DOI:** 10.3390/molecules30122523

**Published:** 2025-06-09

**Authors:** Lin Li, Zhihong Wang, Wei Zhang, Longxin Chen, Yingying Yang, Wan Yang, Mengkun Li, Chunhuan Yuan, Limeng Zhang, Linqing Wang

**Affiliations:** 1 College of Life Sciences, Zhengzhou Normal University, Zhengzhou 450044, China; lilin29pumc@163.com (L.L.); zhangwei19876462@126.com (W.Z.); lxchen@zznu.edu.cn (L.C.); yyy3403@163.com (Y.Y.); yw202209063488@163.com (W.Y.); l2938059365k@163.com (M.L.); ych18461082590@outlook.com (C.Y.); 2 Henan Province Engineering Technology Research Center of Antibody Screening and Diagnostic, Zhengzhou 450044, China; 3 College of Special Education, Zhengzhou Normal University, Zhengzhou 450044, China; wzhcgl@126.com; 4 Laboratory of Molecular Biology, Zhengzhou Normal University, Zhengzhou 450044, China

**Keywords:** *Achyranthes bidentata*, polysaccharides, structural characterization, bioactivity, structure–activity relationships

## Abstract

*Achyranthes bidentata*, a traditional Chinese medicinal herb, has garnered significant attentions due to its diverse bioactivities and substantial market potential. Recent advancements in phytochemical and pharmacological research have identified polysaccharides as some of its primary bioactive constituents. These polysaccharides demonstrate a wide range of biological effects both in vitro and in vivo, such as antioxidant, immunomodulatory, antitumor, anti-inflammatory, anti-osteoporotic, and gut microbiota-regulating properties. This review comprehensively examines the extraction and purification techniques, structural features, modifications, pharmacological effects, and structure–activity relationships of *A. bidentata* polysaccharides (ABPs) over the past three decades. By highlighting the multifaceted biological roles and structure–activity correlations of ABPs, this review aims to enhance the understanding of their potential applications and foster further innovation in bioactive research and development.

## 1. Introduction

*Achyranthes bidentata* Blume belongs to the genus *Amaranthaceae* and is widely distributed in tropical and subtropical regions, particularly in Europe and southern and eastern Asia [1]. As a perennial herb with a cultivation history spanning two millennia in China, *A. bidentata* Blume predominantly thrives in the central and southwestern provinces [2]. Its desiccated rhizomes, pharmacologically designated as “Niuxi” in TCM practice, constitute a classical botanical drug systematically cataloged within the Compendium of Materia Medica, an authoritative Ming Dynasty treatise on ethnopharmacology [3]. The species’ extended domestication period and regional distribution patterns underscore its historical significance in East Asian herbal medicine systems [3,4]. The perennial herb *A. bidentata* has been pharmacologically characterized for its traditional therapeutic applications in hepatic–renal system tonification, musculoskeletal reinforcement, hematological stagnation resolution, and menstrual cycle regulation [2,5]. Contemporary pharmacological investigations have demonstrated that *A. bidentata* exhibits multifaceted therapeutic properties, including suppression of osteoclast differentiation, attenuation of inflammatory responses, viral replication inhibition, immune system regulation, and neoplasm growth restriction [2,6]. These bioactive manifestations are principally associated with the plant’s heterogeneous phytochemical composition, comprising carbohydrate polymers, protein-derived chains, pentacyclic saponins, steroidal ketones, and aromatic essential extracts, among other bioactive constituents [5,7].

Phytochemical analyses have identified polysaccharide fractions (ABPs) as principal pharmacological components within *A. bidentata*, demonstrating intrinsic biocompatibility and multifaceted therapeutic potential [8]. Contemporary research confirms that isolation protocols significantly influence the molecular architecture of these biopolymers, resulting in distinct physicochemical profiles that correlate with specific biological functions such as oxidative stress mitigation, bone density preservation, immunomodulatory capacities, neoplastic proliferation inhibition, and metabolic endurance enhancement [9,10].

Current literature analysis reveals a notable absence of systematic evaluations addressing both isolation methodologies and molecular configurations of polysaccharides derived from *A. bidentata* Blume, along with their associated bioactive potentials. To address this research gap, the present work prioritized a comprehensive integration of refinement protocols for these biopolymers, while critically interrogating the structure–function correlations inherent to their pharmacological efficacy. Such an analytical framework could not only consolidate fragmented technical knowledge but also advance mechanistic interpretations of their therapeutic applications. Moving forward, subsequent investigations should prioritize exploring innovative applications and functional optimization within the realm of polysaccharide-based biomaterials, particularly emphasizing their sustainable production methodologies and structure–activity relationships.

## 2. Data and Methods

### 2.1. Literature Search Strategy

We mainly conducted the systematic literature search in April 2025. The search spanned publications from January 2000 to April 2025, with emphasis on recent pivotal studies published within the preceding ten years, while selectively including seminal earlier works. Two major databases were interrogated: China National Knowledge Infrastructure (CNKI) for Chinese literature and Web of Science for international publications.

### 2.2. Search Terminology

**Figure 1:**
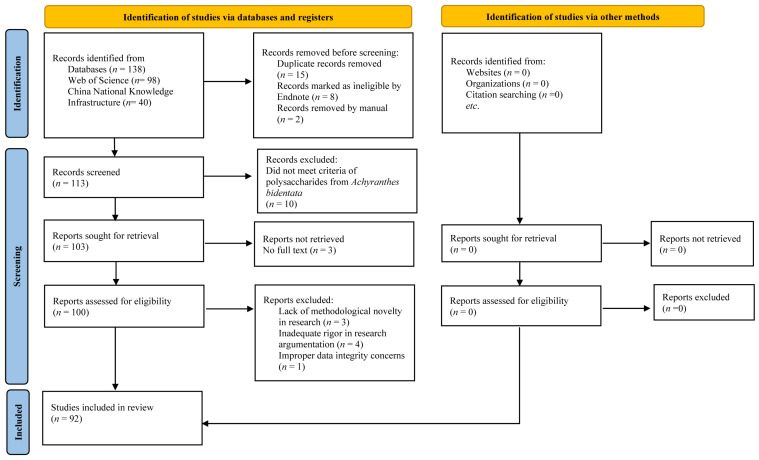
PRISMA literature screening and inclusion flowchart.

Chinese search terms included core concepts: *Achyranthes bidentata* polysaccharides, structure–activity relationship, etc. as subject headings. English searches employed controlled vocabulary and free-text terms combining *Achyranthes bidentata* with methodological descriptors (polysaccharide purification, structural characterization, bioactivity evaluation). The Web of Science search strategy (Figure 1) specifically utilized “polysaccharide of *Achyranthes bidentata*” as a primary topic term.

### 2.3. Inclusion/Exclusion Criteria

Studies addressed either (i) extraction/purification protocols, (ii) structural elucidation (molecular weight, monosaccharide composition, glycosidic linkages), or (iii) demonstrated bioactivities of *A. bidentata* polysaccharides. Priority was given to studies employing contemporary analytical techniques (HPLC, GC, FTIR, GC-MS, NMR, SEM).

Non-relevant publications (*n* = 10), duplicates (*n* = 15), algorithmically flagged ineligible records via Endnote (*n* = 8), manually excluded irrelevant studies (*n* = 2), and unavailable full-text articles (*n* = 3) were sequentially removed. Post full-text review, additional exclusions were made for lack of methodological novelty (*n* = 3), insufficient experimental rigor (*n* = 4), and data integrity concerns (*n* = 1).

### 2.4. Data Synthesis

From an initial pool of 138 records, 92 studies met final inclusion criteria (18 Chinese articles from CNKI; 74 English article from Web of Science). The PRISMA-compliant selection flowchart documents screening stages (Figure 1, Supplementary Materials).

## 3. Extraction and Purification Methods

Polysaccharides derived from *A. bidentata* (ABPs) are primarily localized within the plant cell wall architecture, necessitating extraction strategies that focus on the controlled breakdown of cell wall components [11]. Standard isolation procedures employ mild physicochemical conditions to selectively disrupt the outer lignocellulosic barriers while preserving the structural integrity and functional properties of ABPs (Table 1) [11,12]. This methodological emphasis ensures minimal alteration to the native polysaccharide conformations, thereby maintaining their bioactivity for subsequent pharmacological evaluations. In industrial applications and laboratory settings, hydrothermal extraction has emerged as a preferred technique for polysaccharide isolation due to its operational practicality and enhanced process kinetics [13]. The elevated temperature conditions inherent in this method could significantly improve mass transfer efficiency and solute diffusivity, thereby optimizing the extraction yield. This phenomenon might be attributed to the Arrhenius-type relationship between temperature and molecular mobility in aqueous media, which facilitates the disruption of cellular matrices and subsequent release of bioactive polymers [14]. Contemporary extraction methodologies employing aqueous thermal processing exhibit notable constraints, particularly regarding prolonged operational duration, elevated thermal requirements, suboptimal productivity, and potential structural compromise of active biopolymers (ABPs). To address these limitations, innovative enhancement strategies have been implemented, encompassing sonication-enhanced protocols, electromagnetic wave-assisted techniques, and biocatalytic digestion processes [11]. The sequential procedures for isolating and refining polysaccharide components from *A. bidentata* are systematically outlined in Table 1, encompassing both primary extraction phases and subsequent purification stages.

To enhance the production efficiency of ABPs, multiple extraction methodologies have been systematically investigated. Previous research by Zhang et al. established an aqueous extraction protocol utilizing deionized water as the solvent under optimized parameters: three successive extraction cycles with a 10:1 solvent-to-solid ratio at 80 °C for 3 h per cycle [11]. Subsequent purification through sequential chromatography using DEAE-cellulose and Sephadex columns enabled isolation of two distinct polysaccharide fractions. Comparative analysis revealed that ultrasound-assisted extraction demonstrated superior efficiency, with experimental data indicating optimal performance under specific operational parameters: 60-min processing duration at 60 °C with a 30:1 liquid-solid ratio and 1000 W ultrasonic intensity [15]. Notably, this advanced technique achieved a maximum extraction yield of 6.1% when applied to root specimens collected from Wushe County, Jiaozuo City (Henan Province), significantly surpassing conventional thermal extraction outcomes. The observed enhancement in extraction efficiency could be attributed to the synergistic effects of acoustic cavitation and thermal acceleration inherent to ultrasonic processing technology [16,17]. Modified extraction protocols demonstrated significant improvements in ABPs production through parametric optimization [18]. Experimental data revealed that adjusted thermal conditions (50–60 °C) combined with reduced microwave irradiation intensity (600–800 W) and extended exposure duration (2–4 min) under elevated liquid-to-solid ratios (16:1–30:1) synergistically enhanced polysaccharide recovery, achieving a remarkable yield of 12.37%. Furthermore, enzymatic hydrolysis has emerged as an effective biological approach for polysaccharide isolation. In a systematic investigation by Ye et al., processed parameters were statistically optimized through orthogonal experimental design, establishing optimal operational specifications: 45 °C incubation temperature, pH 6.0 buffer system, and 0.7% (*w*/*w*) tri-enzyme cocktail (cellulase/pectinase/protease at 1:1:1 ratio) with 20:1 aqueous phase-to-biomass proportion. Comparative analysis of these advanced extraction technologies indicated three principal advantages: (1) 38.6% average enhancement in extraction efficiency compared to conventional methods; (2) 40–60% reduction in processing duration through accelerated mass transfer mechanisms; (3) sustainable benefits from 25 to 35% decreased in solvent consumption and energy expenditure. These technological innovations collectively address critical challenges in phytopolysaccharide production, particularly in terms of process intensification and environmental footprint reduction [3].

**Figure 2:**
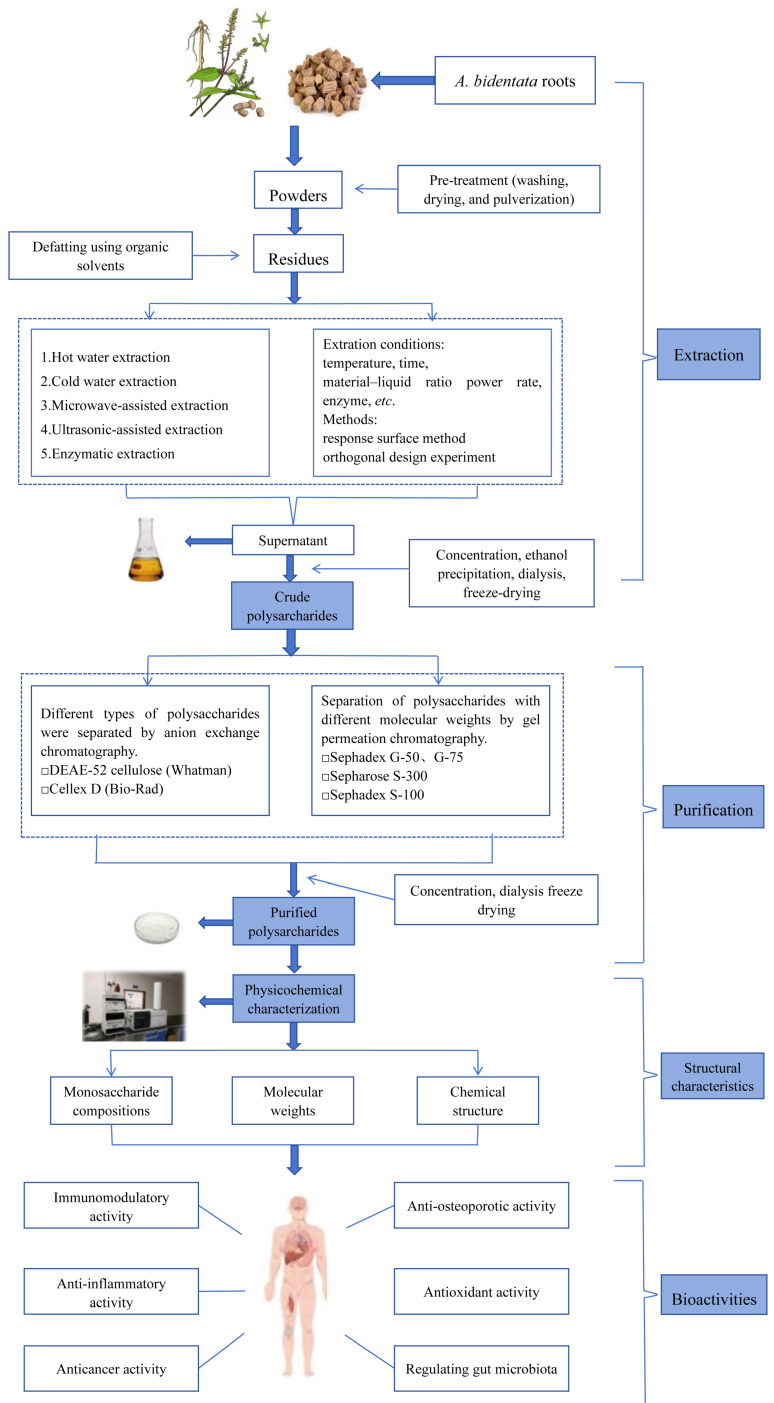
Schematic representation of the extraction and purification of polysaccharides from *A. bidentata*.

The crude ABPs can be further purified by a combination of techniques, including deproteinization, decolorization, ion-exchange chromatography, and gel filtration chromatography (Figure 2) [19]. The figments can be removed by the adsorption of activated carbon and macroporous resin, and protein removed using the Sevag method, the trichloro-trifluoroethane (CCl_2_FCClF_2_) method, the trichloroacetic acid (TCA) method, and enzymic methods. Ion-exchange chromatography differentiates between neutral and acidic polysaccharides by employing NaCl eluents of varying concentrations [20]. Meanwhile, gel filtration is utilized to segregate polysaccharides based on their distinct molecular weights (*Mw*). Wang et al. isolated three polysaccharide fractions (ABP50-2, ABP70-2, and ABP90-2) from *A. bidentata* with DEAE-Cellulose 52 chromatography and Sepharose S-100 chromatography [21]. ABP50-2, ABP70-2, and ABP90-2 contained fructose and glucose in a molar ratio of 27:1, 18:1 and 16:1, respectively. Zhang et al. fractionated ABPs with DEAE-cellulose 52 column (Φ 2.6 × 40 cm) eluted with 0.05, 0.1, 0.15, 0.2 M NaCl solution. The collected fractions were further purified on a gel-filtration column of Sephadex G-75 (Φ 1.6 × 100 cm) with deionized water as the eluent. Fraction ABPB-3 (the acidic polysaccharide) contained arabinose, galactose, rhamnose, and galacturonic acid with a ratio of 18.4:9.3:2.5:1.0 [22].

The isolation and purification of ABPs involve a systematic operational workflow. Fresh roots undergo thorough washing, longitudinal sectioning, and surface processing to remove impurities. Dehydration is performed through thermal drying at 80 °C for 48 h or natural solar desiccation. The dried roots are subsequently pulverized and sifted through 100-mesh sieves to obtain homogenized powder. A preliminary extraction step employs 80% ethanol in a water bath maintained at 80 °C for 2 h to eliminate lipid-soluble components, pigments, and small-molecular-weight saccharides. The defatted residue undergoes aqueous or alkaline-assisted extraction with optimized processing parameters. The resulting polysaccharide-containing solution is subjected to filtration and vacuum concentration to obtain crude extracts. After deproteinization, decolorization, dialysis, evaporation, precipitation with ethanol, filtration, and lyophilization, the crude ABPs are obtained. The purified ABP fractions are obtained through sequential chromatographic purification processes. Crude ABP solutions undergo dissolution and loaded onto specified separation columns, with fractionation achieved using optimized mobile phases. Eluates are systematically fractionated, subjected to membrane dialysis, reduced under vacuum, and finally stabilized through lyophilization. Quantitative analysis of carbohydrate content was performed via the phenol-sulfate colorimetric assay, while protein contaminants were assessed using the Bradford assay with bovine serum albumin as reference standard.

## 4. Physicochemical and Structural Features

Plant polysaccharides are characterized by molecular mass distribution, monosaccharide composition, glycosidic bond configurations (types and positions), and saccharide sequences [23,24]. For ABPs, diverse structural variants have been identified through chromatographic isolation. Structural elucidation of purified ABPs employs integrated techniques: spectroscopic analysis (FT-IR, NMR), chromatography (HPLC, GC-MS), and chemical derivatization (acid hydrolysis, periodate oxidation). As summarized in Table 2, these analyses reveal critical structural parameters including molecular weights (10^3^–10^6^ Da), heterogeneous monosaccharide profiles (glucose/galactose/arabinose), and associated bioactivities. The established structure–function relationships provide molecular insights into ABPs’ pharmacological properties, particularly their immunoregulatory and antioxidant effects.

### 4.1. Average Molecular Weights

Different techniques, including HPLC and high-performance gel permeation chromatography (HPGPC), have been used to determine the average *Mw*s of ABPs, and many studies of ABPs have been based on the same methods. Yan et al. demonstrated that the molecular weights of ABPs (ABPB-3 and ABPB-4) were 77,230 and 63,500 Da, respectively [22,27]. In addition, Chen et al. obtained three polysaccharides by Sephadex G-50 and CM-Sephadex C-50 column. The *Mw* of CoPS1, CoPS2, and CoPS3 were 5200, 3000, and 1400 Da, respectively, through HPLC [29]. Under diverse experimental conditions and across various *A. bidentata* polysaccharide samples, molecular weights within the 10^4^–10^5^ Da range were detected, presenting different values.

### 4.2. Monosaccharide Compositions

Phytogenic polysaccharides inherently possess structural heterogeneity, manifested through varied monosaccharide compositions and distinct molecular configurations [35,36]. The structural polymorphism observed in ABPs principally originates from differential processing parameters, particularly solvent systems employed during isolation, separation techniques utilized for fractionation, and refinement procedures implemented for molecular purification. In addition, the difference of monosaccharide composition and connection mode could be also one of the reasons for the diversity of polysaccharide structures. Therefore, comprehensive monosaccharide composition analysis and structural–activity relationship elucidation of ABPs are indispensable steps. Structural characterization of ABPs typically employs pre-chromatographic derivatization integrated with chromatographic and spectrometric analytical systems, including high-performance liquid chromatography coupled with gas chromatography–mass spectrometry. Most ABPs were mainly composed of fructose (Fru), glucose (Glc), galactose (Gal), galacturonic acid (GalA), glucuronic acid (GlcA), arabinose (Ara), and rhamnose (Rha) being rare and exhibiting variability in different molar ratios [11,19]. Different extraction environments could influence the monosaccharide composition of ABPs. Yan et al. obtained three polysaccharides, namely ABW50-1, ABW70-1, and ABW90-1, through alcohol precipitation with different concentrations of ethanol and found that three ABPs had different monosaccharide (Fru and Glc) ratios [2,11,26]. A new peptide polysaccharide (ABAB) was purified by Cellex D and Sephadex G-150 column, which was composed of GlcA, Gal, GalA, Ara, and Rha with a molar ratio of 12:2:3:1:1 [32]. Active ABPs displayed significant variability in monosaccharide combinations with distinct quantitative proportions. Intraspecific specimens from diverse cultivation areas frequently exhibited notable variations in their monosaccharide compositions.

### 4.3. Chemical Structures

**Figure 3:**
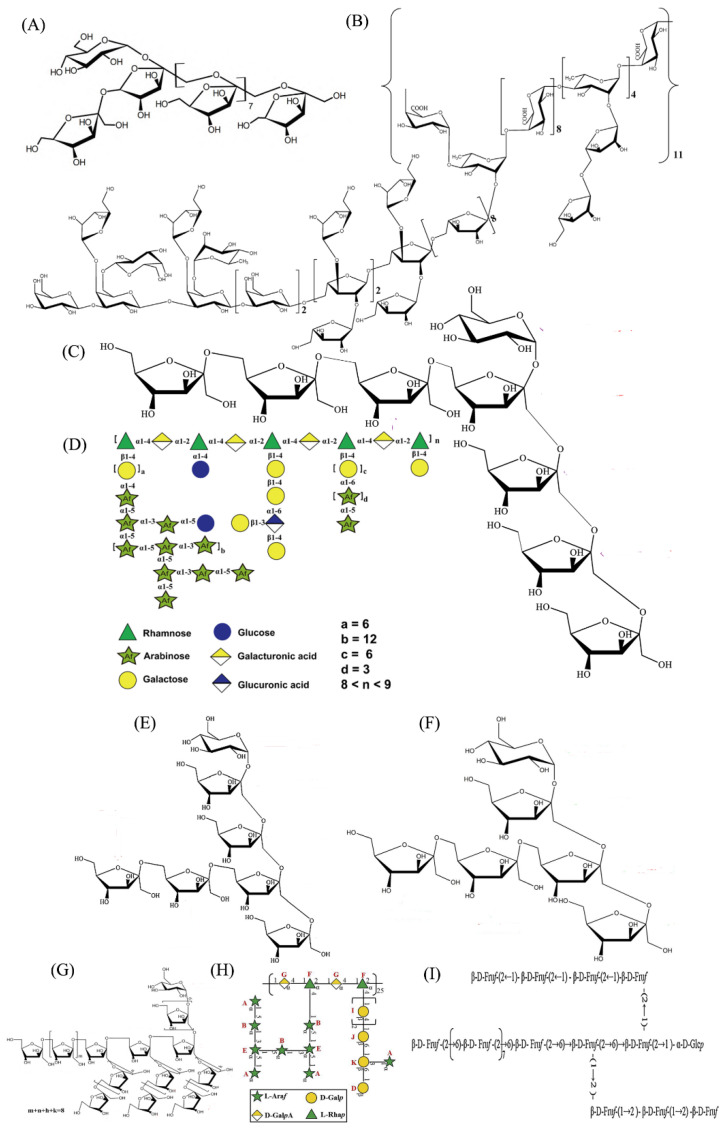
Schematic structures of *A. bidentata* polysaccharides. (**A**) ABP [31], (**B**) ABPB-3 [22], (**C**) ABW70-1 [11], (**D**) A23-1 [19], (**E**) ABW50-1 [25], (**F**) ABW90-1 [26], (**G**) ABPW1 [28], (**H**) ABPB-4 [27], (**I**) ABP70-2 [21].

Contemporary research has characterized numerous structural variants of ABPs, where isolation and refinement protocols critically determine their molecular configurations. Comprehensive elucidation of plant polysaccharide architectures employs advanced analytical methodologies including gas chromatography–mass spectrometry, methylated derivative analysis, infrared spectral characterization, and nuclear magnetic resonance spectroscopy [37,38]. The current inferred data on the repeat units of ABPs are shown in Figure 3. An early publication in 1990 first reported that purified *A. bidentata* polysaccharide (ABAB) contained glucuronic acid, galactose, galacturonic acid, and arabinose, with the backbone including →1)-d-Glc*p*A-(4→ and →4)-d-Gal*p*A-(1→ [32]. In another study mentioned above, the structural features of a water-soluble polysaccharide (M2) with hypoglycemic activity were investigated by MS analysis and ^1^H, ^13^C NMR spectroscopy. The primary M2 structure was deduced to be a fructooligosaccharide consisting of α-d-Glc*p*-(1→ and →1)-Fru*f*-(2→ backbone with substitution at *O*-6 on different residues [30].

In addition, the structure of ABP70-2 with a lower *Mw* of 3406 Da was determined by monosaccharide analysis, infrared spectroscopy, methylation, gas chromatography–mass spectrometry (GC-MS) analysis, and NMR studies (^1^H, ^13^C, NOESY, HSQC, and HMBC). It emerged that the backbone of ABP70-2 possessed a backbone of (2→6)-linked *β*-d-Fru*f*, with (2→1)-linked *β*-d-Fru*f* branched chains, and terminated with Glc*p* and Fru*f* residues [21]. Water-soluble pectin A23-1 was investigated with GC-MS, partial acid hydrolysis, and one-dimensional (^1^H NMR and ^13^C NMR) and two-dimensional (HSQC, ^1^H-^1^H COSY and HMBC) spectra. The backbone of A23-1 was linearly connected by 1,2,4-Rha*p* and 1,4-Gla*p*A, and three different branches were composed of galactose, arabinose, and glucose, which linked at *C*-4 of rhamnose, respectively [19].

The primary structures of ABPB-3 and ABPB-4 were determined with a combination of chemical and instrumental analyses, including methylation analysis, GC, FT-IR, and NMR. ABPB-3 was mainly composed of →4)-α-d-Gal*p*A-(1→, →2,4)-α-l-Rha*p*-(1→, →5)-α-l-Ara*f*-(1→, →2,3,5)-α-l-Ara*f*-(1→, →3)-*β*-d-Gal*p*- (1→, →3,4,6)-*β*-d-Gal*p*-(1→; ABPB-4 was composed of →4)-α-d-Gal*p*A-(1→ and →2,4)-α-l-Rha*p*-(1→ (22,27).

### 4.4. Conformational Features

The biofunctional characteristics of plant polysaccharides correlate with critical physicochemical parameters including molar mass parameters, structural complexity, and tertiary structural arrangements [39,40]. Current understanding indicates significant research gaps regarding the solution-phase characteristics and supramolecular configurations of ABPs. While Wang’s team preliminarily investigated helical conformations in specific ABP fractions, subsequent analyses confirmed the absence of triplex helical conformation in the ABP70-2 fraction through circular dichroism analysis [21]. Moreover, the SEM could display the spatial distribution, arrangement, and interactions with other molecules of plant polysaccharides. ABW70-1 exhibited an irregular granular shape with small holes internally [11], and ABW90-1 was mainly composed of randomly distributed, sheet-like appearances [26]. ABPB-3 was fragmented with a honeycomb-like morphology [22]; however, ABPB-4 was composed of irregular sheets with obvious branches and smooth surfaces [27].

Determining the relationships between the chain conformations and solution properties of ABPs and their corresponding biological activities remains challenging. To gain insights into the detailed chain conformations of ABPs in aqueous solutions, the employment of advanced techniques is necessary. These techniques include viscosity measurements, static and dynamic light scattering, circular dichroism, transmission and atomic force microscopy, fluorescence spectrometry, and NMR spectrometry, all of which could contribute to further investigations [20,41].

### 4.5. Structural Modification

Current modification strategies for ABPs could enhance bioactivity through chemical derivatization, physical processing, and enzymatic biotransformation. ABPs caused by the steam explosion pretreatment could significantly increase their antioxidant activities in vitro and in vivo [42]. The sulfated and sulfated-esterification modification could enhance antiviral activities in vitro of ABPs [43]. The carboxymethyl groups could substitute on the 4-position of the fructofuranose of the backbone and obviously improve the antitumor activity of ABPs [44]. The phosphorylation ABPs could increase the weight of diabetes rats, reduce the levels of serum TC, TG, and LDL-C, and significantly increase the level of HDL-C [45]. The above studies have shown that the biological activity of ABPs could be significantly enhanced by different modification methods [3]. The specific mechanisms of ABPs of action different modification methods on biological functions still needs to be further studied.

## 5. Biological Activities

In traditional Chinese medical practice, the rhizome of *Achyranthes bidentata* has been historically employed as a therapeutic agent for blood circulation enhancement and stagnation alleviation. Clinical applications encompass multiple physiological systems, particularly addressing (1) gynecological disorders (menstrual irregularities and dysmenorrhea), (2) musculoskeletal conditions (lumbar strain and joint inflammation), (3) urinary system abnormalities (edema and dysuria), along with neurological manifestations (cephalalgia and vertigo) and hemorrhagic symptoms (hematemesis and nasal bleeding) [2,3,30]. Contemporary phytochemical investigations have identified polysaccharide complexes as principal bioactive constituents underlying its ethnopharmacological efficacy [3]. Accumulating evidence suggests these macromolecules could mediate diverse therapeutic outcomes through immunomodulatory, anti-inflammatory, and cellular protective mechanisms. The subsequent analysis systematically evaluates the structure-dependent, biofunctional properties of ABPs, providing crucial insights into their pharmacodynamic basis.

### 5.1. Antioxidant Activity

**Figure 4:**
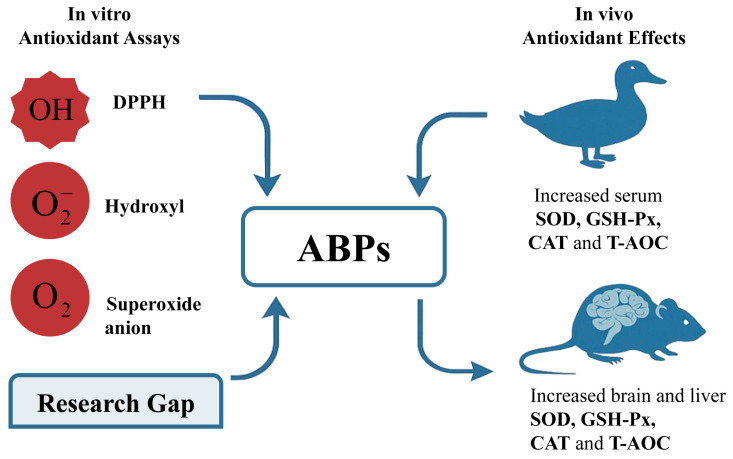
Schematic diagram of ABP function in antioxidant activity.

Natural substances hold great promise as antioxidant sources. Plants, fungi, and animals contain diverse bioactive components, among which polysaccharides particularly exhibit antioxidant properties [9,46]. Antioxidant capabilities have been central to numerous studies exploring the nutraceutical and therapeutic mechanisms of traditional Chinese medicines, relying on multiple assay techniques and activity metrics [47,48]. Prior research has shown that ABPs possess potential antioxidant activity, and this effect has a positive correlation with the ABPs’ concentration (Figure 4). Many research groups have demonstrated the antioxidative activities of ABPs in vitro and in vivo [3]. ABP intervention could increase serum superoxide dismutase, glutathione peroxidase, total antioxidative capacity, and catalase in Pekin ducks [49]. ABPs also could increase the level of total antioxidant capacity and the activity of total superoxide dismutase, catalase, and glutathione peroxidase in the brain and liver tissues of d-galactose-induced aging-model mice [50]. Wei et al. recently demonstrated that ABPs through ultrasonic-assisted extraction had definite antioxidative activity, estimated in 2,2-diphenyl-1-picrylhydrazyl radical, hydroxyl radical, and superoxide anion systems [15]. ABPs displayed a dose-dependent DPPH-radical-scavenging effect of 75.1%, a hydroxyl-radical-scavenging effect of 90.3%, and a superoxide-anion-scavenging effect of 61.3% at the tested concentration of 12 mg/mL. Additional investigations are needed to explore the underlying mechanisms [51]. Conducting more in vivo trials is essential to validate the utilization of ABPs in the area of antioxidant-based functional foods.

### 5.2. Immunomodulatory Activity

Immune regulation mechanisms constitute fundamental defense processes capable of identifying and eliminating exogenous threats through biological recognition systems [52]. Plant-derived polysaccharides could exhibit bidirectional immunoregulatory properties, capable of modulating host defense responses through either potentiation or attenuation pathways [53,54]. Notably, ABPs demonstrate immunoenhancing effects via multi-target activation of innate and adaptive immune components: stimulation of phagocytic macrophages, proliferation of T lymphocytes, maturation of dendritic cells, and cytotoxic natural killer cell activation [55,56]. Concurrently, these biopolymers enhance humoral immune responses through splenocyte proliferation potentiation and antigen-specific antibody production elevation [57,58]. Fan et al. reported that treatment with ABPs at doses of 200, 500, and 1000 μg/mL significantly increased the secretion of IL-1β, TNF-α, and NO in J744 A.1 cells and decreased the activation of Toll-like receptor 4 (TLR4) antibody and CD14/TLR4 antibody. The mRNA and protein expression of TLR4 and MyD88 were also significantly increased after ABPS treatment. The immunomodulatory mechanism of ABPs might be associated with the secretion of cytokines by stimulating the NF-κB pathway through TLR4/MyD88 signaling [59].

The immunomodulatory activities of ABPs were previously investigated by Lei et al. in cyclophosphamide-induced rats. Their results indicated that ABPs could significantly improve the spleen index and alleviate the pathological changes in immune organs and increase the levels of IL-2, IL-6, IFN-γ, and TNF-α. The transcription of GATA-3, Foxp3, and ROR were decreased, while T-bet was increased. Among the differentially expressed genes, Th2-related genes were notably enriched. Representative genes such as Il4, Il13, and Il9 were included. Concurrently, the expression of immune effector genes like Ccl3, Ccr5, and Il12rb2 was elevated. ABPs likely modulated the cytokine balance in helper T cells, thereby improving the immune function of cyclophosphamide-induced mice [4].

**Figure 5:**
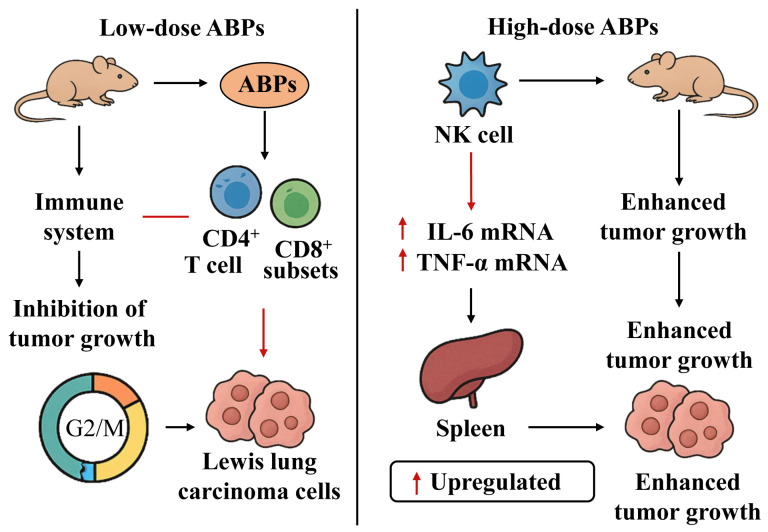
Schematic diagram of ABPs’ function in immunomodulatory activity.

Nutritional supplementation with ABPs demonstrated immunostimulatory effects in weaned piglets, augmenting both systemic and mucosal immune modulation [60]. In vitro studies further revealed that ABPs-enriched culture media significantly elevated lymphocyte proliferative capacity and cytokine synthesis efficiency (Figure 5) [61]. Notably, structure–activity relationships were observed wherein distinct modification strategies of ABPs selectively enhanced the secretory profiles of targeted immunoregulatory molecules, suggesting methodology-dependent immunomodulatory mechanisms.

### 5.3. Anti-Osteoporosis Activity

Osteoporosis and osteoporosis-related fractures are major public health problems worldwide [62,63]. It has been found that ABPs exhibit anti-osteoporotic effects [3]. Osteoclasts play a fundamental role in bone resorption and the pathogenesis of osteolytic conditions. AB70 exerted osteoprotective effects on bone mass, which was manifested through enhancing trabecular bone microstructure and reducing the serum concentrations of biomarkers related to bone turnover [64]. ABW70-1 stimulated the osteogenic differentiation of MC3T3-E1 cells by promoting cell proliferation, ALP activity, mineral nodule formation and the gene expression of *Osx*, *Ocn*, and *Bsp* [11].

**Figure 6:**
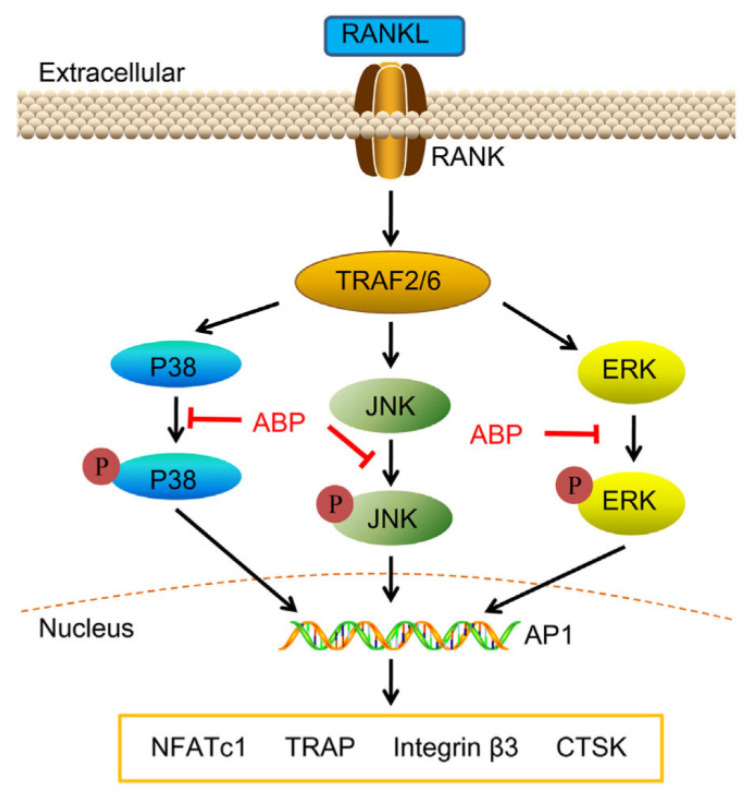
Schematic diagram of ABPs’ function in anti-osteoporosis activity [66].

Chronic AB90 supplementation via oral gavage demonstrated osteoprotective efficacy, effectively preserving bone mineral density and enhancing skeletal biomechanical integrity in preclinical models. AB90 treatments significantly suppressed the ovariectomized-induced increases of serum OCN, PINP, and CTX, indicating that AB90 was positive in attenuating the bone turnover and resorption in ovariectomized rats [26]. As shown in Figure 6, ABPs could decrease the expression of NFATc1, Integrin β3, TRAcP, and CTSK via suppressing the phosphorylation of MAPK pathways, which inhibited RANKL-induced osteoclast differentiation and function [65,66].

ABPB-3 demonstrated dose-dependent enhancement in skeletal fluorescence signal intensity, suggesting its pharmacodynamic properties in activating osteogenic processes [22]. Furthermore, ABPB-4 exhibited pronounced stimulatory effects on the proliferation capacity, differentiation potential, and mineral deposition of MC3T3-E1 pre-osteoblasts in vitro, accompanied by upregulated transcriptional regulation of key osteogenic differentiation markers [27]. Nevertheless, current understanding of the molecular mechanisms governing their anti-osteoporotic efficacy remains incomplete, necessitating systematic investigation into the fundamental biological processes mediating these therapeutic outcomes.

### 5.4. Antitumor Activity

**Figure 7:**
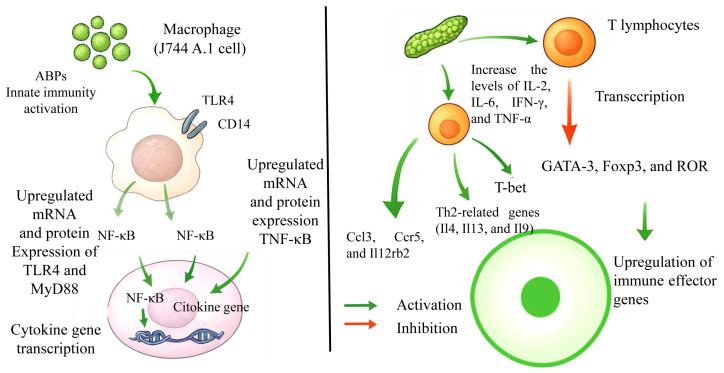
Schematic diagram of ABPs’ function in antitumor activity.

The antitumor activity of plant polysaccharides is affected by the size of the molecules, their form, degree of branching, and solubility in water [67]. Generally, the greater the molecular weight and the higher the water solubility of the polysaccharide, the greater is its antitumor activity [68]. Accumulating evidence indicates that bioactive polysaccharides manifest potent tumor-suppressive properties via distinct molecular pathways [20]. The established mechanisms underlying these biopolymer-mediated anticancer actions could be systematically categorized into four principal modes: (1) chemopreventive effects through gastrointestinal absorption, (2) immunomodulatory enhancement of host antitumor surveillance, (3) cytotoxic induction of programmed cell death pathways, and (4) inhibition of metastatic dissemination through extracellular matrix modulation [69,70]. To clarify the precise antitumor mechanisms of ABPs (Figure 7), Jin et al. reported the investigation on the effects of ABPs against Lewis lung cancer (LLC) in C57BL/6 mice. ABPs at low doses could significantly inhibit LLC growth; tumor cells arrested at the G2/M phase after daily low-dose intraperitoneal injection of ABPS for 15 consecutive days. At low dosage, ABPs were capable of inhibiting tumor growth. They achieved this by inducing the arrest of tumor cells in the G2/M phase [71]. In contrast, when at a high dosage, ABPs promoted tumor growth. This occurred through the impairment of NK cell function and the upregulation of the expression of IL-6 mRNA and TNF-α mRNA within the spleen. Cao et al. investigated the effects of ABPs on T cell subsets, vascular endothelial growth factor (VEGF), and tumor growth transforming factor-*β*_1_ (TGF-*β*_1_) in the tumor microenvironment of HepG-2 tumor-bearing mice. Experimental data revealed that ABP administration markedly suppressed the proliferative capacity of neoplastic cells while concomitantly enhancing the immunophenotypic profile through elevation of CD4+/CD8+ T cell subsets. Quantitative analysis further demonstrated significant downregulation in protein expression levels of pro-angiogenic factors (VEGF) and fibrogenic mediators (TGF-*β*_1_) within the tumor microenvironment [72,73].

### 5.5. Anti-Inflammatory Activity

The anti-inflammatory mechanism of plant polysaccharides involves several key aspects: they could influence the production of TNF-α, IL-1*β*, IL-6, and other cytokines; regulate the activity of immune cells such as macrophages, B cell, T cell, and other immune cells; address oxidative stress, which is a key cause of inflammation, with antioxidant activity also reducing inflammation; and affect signaling pathways involved in the inflammatory response [74,75]. ABPs could inhibit thapsigargin (TG)-induced chondrocyte ERS through the lncRNA NEAT1/miR-377-3p axis and improve the ankle joint destruction in rheumatoid arthritis rats by downregulating the MTA1/NF-κB pathway [76]. In an experiment of acute gouty arthritis in rats, ABPs effectively inhibited the swelling degree of arthritis, suppressed joint flexion and extension pain, and released serum inflammatory factors [30,77].

### 5.6. Regulating Gut Microbiota

The intestinal tract is the primary site for the absorption of vitamins, minerals, and other nutrients, while the intestinal flora is crucial in maintaining immune function and preventing various diseases [14,78]. As carbohydrates that could not be digested and absorbed by the human body, natural plant polysaccharides are utilized by beneficial microorganisms in the intestine, thus influencing the intestinal flora [79,80]. For example, short-chain fatty acids can be produced, selectively promoting the growth and colonization of certain beneficial flora, inhibiting the proliferation of harmful flora, and binding to receptors on the surface of intestinal cells to trigger intracellular signaling pathways [81]. This, in turn, affects the metabolism and function of cells.

ABPs could enhance intestinal morphology and functional impairments, improve the integrity of the intestinal barrier, adjust the composition of the intestinal microbes, promote the production of short-chain fatty acids, and inhibit the production of intestinal inflammation [19]. The levels of short-chain fatty acids, particularly acetic acid, propionic acid, and butyric acid, increased after the ABPs were fermented by human fecal microorganisms in vitro [28]. A diet supplemented with ABPs significantly enhanced the *Firmicutes*/*Bacteroidetes* proportion and selectively enriched *Ruminococcaceae* and *Lachnospiraceae* taxa. This nutritional intervention induced structural reorganization of cecal microbiota ecosystems [82]. Additionally, ABP-fortified regimens augmented body weight gain parameters, enhanced villus–crypt architectural development, and restored enteric microbial homeostasis in broilers subjected to pathogenic *E. coli* K88 infection [83]. ABPs played a hypoglycemic role by increasing gut microbiota-derived SCFA levels and activating the GLP-1/GLP 1R/cAMP/PKA/CREB/INS pathway [81].

### 5.7. Other Bioactivities

ABPs have the potential to indirectly counter viral infection. This was achieved by modulating the release of immune-related factors like nitric oxide (NO) and interleukin-4 (IL-4). Moreover, sulfated modification could augment the antiviral capabilities of ABPs [84]. Immunol-modulatory ABPs, when used for pretreatment, selectively promoted Th1 immune responses. This promotion effectively regulated the proliferation of malaria parasites. As a result, during subsequent malaria infections, the survival time of mice was extended [85]. Furthermore, ABP intervention exhibited significant anti-fatigue properties, evidenced by prolonged forced-swim endurance. Mechanistically, this regimen elevated hepatic and muscular glycogen reserves while reducing circulating lactate and urea nitrogen concentrations in challenged animals [86]. Supplementation of ABPs increased plasma concentrations of hormones, antibodies, and alkaline phosphatase and IL-1β mRNA abundance in the liver, jejunal mucosa, and lymph nodes and indicated that ABPs were effective in improving growth performance and defending capacity. The 300 mg/kg ABPs significantly reduced the immune liver injury model in mice, and the elevated levels of ALT in serum and liver tissue inhibited the elevated levels of MDA and SOD activity in liver homogenate and reduced the weight index of the spleen and thymus [87]. ABPs had a protective effect on CCl_4_-induced acute liver injury in rats, and the mechanism was believed to be related to ABPs’ anti-lipid peroxidation and anti-inflammatory responses [88]. Experimental investigations revealed that ABP administration demonstrated therapeutic efficacy in ameliorating respiratory parameters and attenuating pro-inflammatory mediators through modulation of the TGF-*β*_1_/Smad signaling axis in rodent models of chronic obstructive pulmonary disease [82]. Furthermore, these bioactive compounds exhibited substantial reduction in eosinophil infiltration and mast cell infiltration within pulmonary tissues, suggesting potential therapeutic interventions for allergic airway inflammation [89]. Notably, systematic investigation into the mechanistic basis of these pharmacodynamic effects, coupled with translational research bridging preclinical findings to clinical validation, remains imperative for comprehensive understanding of their therapeutic potential.

## 6. Correlation of Structure, Content, and Biological Activity

Extensive research efforts have been devoted to developing purification protocols and elucidating the physicochemical properties of bioactive ABPs, with substantial documentation of their pharmacological properties. Nevertheless, critical knowledge gaps persist in deciphering the structure–pharmacodynamics correlations underlying these biopolymers. Current consensus in glycobiology posits that the multifaceted therapeutic effects of plant-derived polysaccharides arise from specific structural determinants, including saccharide sequencing patterns, supramolecular assemblies, and post-isolation derivatizations, which collectively dictate their molecular interactions with biological targets [90,91]. The biological activities of ABPs need to be further discussed, including the effects of relative *Mw*, monosaccharide composition, glycosyl bond type, branching degree, and conformation.

The *Mw* of ABPs has a significant effect on their biological activity. The relationship between *Mw* and the biological activity of ABPs demonstrates non-linear complexity. While antitumor efficacy typically enhances progressively with elevated *Mw* in ABP systems, contrasting evidence reveals inverse correlations between polysaccharide chain length and therapeutic potency in certain plant-derived macromolecules [71]. Four distinct polysaccharide fractions (Abnp1001, Abnp1002, Abap1001, and Abap1002) possessing liver-protective properties were purified from *Agaricus bosporus*. Notably, the lower-*Mw* fractions Abnp1002 and Abap1002 demonstrated superior capacity in reducing ALT and AST serum markers in carbon tetrachloride-damaged murine livers compared to their higher-*Mw* counterparts (Abnp1001 and Abap1001) [92]. These findings emphasize the critical need for systematic investigation of structure–function correlations to advance ABPs development for therapeutic applications.

Structural characterization of plant-derived polysaccharides fundamentally requires comprehensive monosaccharide compositional profiling. Current research indicates that bioactive ABPs predominantly contained glucose and fructose as primary sugar constituents, suggesting these hexose ratios might critically influence pharmacological potency. Notably, three chromatographic fractions (ABW50-1, ABW70-1, and ABW90-1) purified from *A. bidentata* demonstrated identical carbohydrate building blocks through advanced analytical techniques yet displayed differential bioactivity patterns attributable to distinct molar proportion arrangements [11,25,26]. Studies of osteoporosis showed that Fru*f*-rich ABW70-1 (Fru:Glc = 7:1) showed high anti-osteoporotic activity, which might be attributed to the higher fructose content [11]. The uronic acids in ABPs are crucial for their biological activities, and fractions richer in galacturonic acid have higher anti-osteoporosis activities. Yan et al. reported that ABPB-3 and ABPB-4, which contained greater amounts of uronic acids, had the strongest osteogenic activity in vitro [22,27].

The pharmacological properties of botanical polysaccharides are critically influenced by three key structural elements: stereochemical configurations of glycosidic bonds, branching patterns, and three-dimensional molecular arrangements. These hierarchical structural characteristics mediate biofunctional efficacy through mechanisms including target recognition specificity, membrane interaction capacity, and metabolic stability. The therapeutic potential of ABPs stems from distinctive structural motifs, particularly *β*-(1→2) and (1→2,6)-Fru*f* linkages within their backbone configurations, which mediate microbial shifts characterized by elevated *Fimicutes*/*Bacteroidetes* balance and proliferation of specific taxa, including *Ruminococcaceae*/*Lachnospiraceae*, thereby underpinning their osteoprotective efficacy, intestinal microbial modulation, and oxidative stress mitigation capacities [25,26,27,28]. The activity of plant polysaccharides could be influenced by structural alterations. For instance, when ABPs undergo carboxymethyl modification, their antitumor and antioxidant functions are remarkably enhanced [44].

Deciphering the structure–activity correlation of ABPs presents considerable challenges. Current investigations reveal that molecular weight parameters do not exhibit characteristic nonlinear correlations with pharmacological responses. Specifically, structural domains containing precisely 26 monosaccharide units demonstrate decisive regulatory effects on ABPs functionality, where fructose-containing glycosidic linkages exhibit preferential enhancement of therapeutic outcomes in bone density preservation and oxidative stress mitigation [21]. These findings underscore the necessity for systematic investigations into the multidimensional structural determinants governing ABPs’ efficacy, particularly the hierarchical organization of saccharide motifs and their dynamic interactions with biological targets.

## 7. Conclusions and Perspectives

The *Achyranthes bidentata* Blume is a plant used in traditional Chinese medicine and food that has a history of thousands of years. It was regarded as a treasure for soreness and weakness in the waist and knees, as well as muscle and bone weakness in *Shennong Bencao Jing*. In modern times, it has been used to produce a variety of functional foods. At present, researchers have focused on extraction, separation and purification, structural characterization, and biological activity of *Achyranthes bidentata* polysaccharides. The results showed that different extraction methods might affect the extraction rate and even the structure of ABPs. Therefore, combining the extraction methods to improve the extraction efficiency, reduce the cost, and control the change of polysaccharide structure is necessary. In addition, most current research is conducted in the laboratory, leaving significant potential for developing extraction and purification methods suitable for process production.

Most existing studies focus on the structural characterization of ABPs, including the determination of molecular weight, monosaccharide composition, functional groups, and glycosidic bonds. However, few studies have explored their mechanisms of action or structure–activity relationships with other active ingredients. Therefore, using modern analytical instruments to further analyze the structures of ABPs and study their structure–activity relationship and mechanisms of action is the main direction of future research.

The experiments on the biological activities of ABPs have focused on antioxidant and anti-osteoporosis aspects, and the studies on intestinal microorganisms are limited. Further research is needed to investigate the effects of ABPs and their metabolites on intestinal flora, as well as the underlying mechanisms. In addition, the existing activity studies have employed in vitro experiments, cell experiments, and mouse models, and few practical clinical applications have been presented. Future investigations should prioritize the application of multi-omics approaches to elucidate the pharmacological properties and molecular mechanisms of ABPs at the genetic and molecular levels. This systematic analysis could establish a scientific foundation for optimizing the sustainable utilization of *A. bidentata* resources through integrated genomic, proteomic, and metabolomic characterization. Particularly, omics-driven strategies should focus on decoding the biosynthetic pathways and regulatory networks of ABPs by analyzing gene expression patterns, protein interactions, and metabolic pathways, thereby facilitating the development of novel therapeutic agents and functional products derived from this medicinal plant.

## Figures and Tables

**Table 1:** Advantages and disadvantages of different extraction methods and polysaccharide yield.

No.	Method	Advantages	Disadvantages	Extraction Method	Yield	Ref.
1	Hot water extraction	cost-effective, economically viable, minimal impact on polysaccharide structure	considerable impurity content, susceptibility to degradation, prolonged extraction duration, and limited extraction efficiency	three successive extraction cycles with a 10:1 solvent-to-solid ratio at 80 °C for 3 h per cycle	49.18% /29.4%	[11]
2	Cold water extraction	solvent-free, thermal stability, bioactivity retention, suitable for heat-sensitive compounds	low extraction efficiency, time-consuming, limited solubility, poor selectivity	a duration of 5 h, solid-to-liquid ratio of 1:40 (mL/g)	-	[12]
3	Microwave-assisted extraction	elevated yield, short processing duration, minimal impurity introduction	economically unfavorable, challenging for industrial application	a solid-to-liquid ratio of 1:20 (g/mL), microwave power of 300 W, extraction time of 10 min, and temperature of 50 °C	8.2%	[14]
4	Ultrasonic-assisted extraction	capable of preserving structural integrity, user-friendly and low-risk process, enhanced extraction performance	depolymerization of soluble polysaccharides	ultrasonic power of 1000 W, extraction temperature of 60 °C, extraction time of 60 min, a solid-to-liquid ratio of 1:30 (g/mL)	6.1%	[15]
5	Enzyme-assisted extraction	high affinity for target analytes, time-efficient extraction, impurities can be readily eliminated	enzyme deactivation, equipment-intensive process	solid-to-liquid ratio of 1:20 (g/mL), 0.7% compound enzyme addition, pH 6.0, temperature of 45 °C	-	[16]

**Table 2:** The polysaccharides isolated from *A. bidentata*.

No.	Name	Purification	Molecular Weight (Da)	Monosaccharide Composition	Structural Features	Bioactivities	Ref.
1	A23-1	DEAE-Cellulose 52, Sepharose S-300 column	93,085	Rha, GlcA, GalA, Glc, Gal, Ara in a molar ratio of 7.26:0.76:5.12:2.54:23.51:60.81	Backbone of 1,2,4-Rha*p* and 1,4-Gla*p*A, and different branches composed of galactose, arabinose, glucose which were linked at *C*-4 of rhamnose	Regulating gut microbiota	[19]
2	ABW50-1	DEAE-Cellulose 52, Sepharose S-100 column	1260	Fru, Glc in the ratio of 6:1	Backbone of →2)-*β*-d-Fru*f*-(1→, →2)-*β*-d-Fru*f*-(1,6→ and →2)-*β*-d-Fru*f*-(6→, terminated with Glc*p* and Fru*f* residues	Stimulating bone formation	[25]
3	ABW70-1	DEAE-Cellulose 52, Sepharose S-100 column	1316	Fru, Glc in the ratio of 7:1	Backbone of (2→1)-linked-*β*-d-Fru*f*, (2→6)-linked-*β*-d-Fru*f* and (1→1,6)-linked -*β*-d-Fru*f* residues, and terminated with fructose and glucose residues	Anti-osteoporosis	[11]
4	ABW90-1	DEAE-Cellulose 52, Sepharose S-100 column	1074	Fru, Glc in the ratio of 5:1	Backbone of (2→6)-linked *β*-d-Fru*f*, (2→1)-linked *β*-d-Fru*f* residues and terminated with Glc*p* and Fru*f* residues	Anti-osteoporosis	[26]
5	ABPB-3	DEAE-Cellulose 52, Sephadex G-75 column	77,230	Ara:Gal:Rha:GalA in the ratio of 18.4:9.3:2.5:1.0	Backbone of →4)-α-d-Gal*p*A-(1→, →2,4)-α-l-Rha*p*-(1→, →5)-α-l-Ara*f*-(1→, →2,3,5)-α-l-Ara*f*-(1→, →3)-*β*-d-Gal*p*-(1→, →3,4,6)-*β*-d-Gal*p*-(1→, terminated with α-l-Ara*f*, α-l-Rha*p* and *β*-d-Gal*p*	Anti-osteoporosis	[22]
6	ABPB-4	DEAE-Cellulose 52, Sephadex G-75 column	63,500	Rha, Gal, GalA, Ara	Backbone of and →2,4)-α-l-Rha*p*-(1→, and the branch chains →4)-*β*-d-Gal*p* -(1→, →6)-*β*-d-Gal*p*-(1→, →3,6)-*β*-d-Gal*p*-(1→, →5)-α-l-Ara*f*-(1→ and →3,5)-α-l-Ara*f*-(1→, and terminated with α-l-Ara*f*-(1→ and *β*-d-Gal*p*-(1→	Osteogenic activity	[27]
7	ABPW1	DEAE Bestarose FF, Sephadex G25 column	3998	Fru, Glc in the ratio of 24:1	Backbone of →1)-*β*-d-Fru*f*-(2→, →2)-*β*-d-Fru*f*-(6→, →1,6)-*β*-d-Fru*f*-(2→ connected fructose and terminated with T-α-d-Glc*p* and *β*-d-Fru*f*-(2→	Regulating gut microbiota	[28]
8	ABP70-2	DEAE-Cellulose 52, Sephacryl S-100HR column	3406	Fru, Glc in the ratio of 18:1	Backbone of (2→6)-linked *β*-d-Fru*f*, with (2→1)- Linked *β*-d-Fru*f* branched chains, and terminated with Glc*p* and Fru*f* residues	Antioxidant	[21]
9	CoPS1	Sephadex G-50, CM-Sephadex C-50	5200	Fru, Glc in the ratio of 39:1	→2)-Fru*f*-(6→, →1)-Fru*f*-(2→, Fru*f*-(2→, →1,6)- Fru*f*-(2→, α-d-Glc*p*-(1→		[29]
10	CoPS2	Sephadex G-50, CM-Sephadex C-50	3000	Fru, Glc in the ratio of 36:1	→2)-Fru*f*-(6→, →1)-Fru*f*-(2→, Fru*f*-(2→, →1,6)- Fru*f*-(2→, α-d-Glc*p*-(1→		[29]
11	CoPS3	Sephadex G-50, CM-Sephadex C-50	1400	Fru, Glc in the ratio of 21:1	→2)-Fru*f*-(6→, →1)-Fru*f*-(2→, Fru*f*-(2→, →1,6)- Fru*f*-(2→, α-d-Glc*p*-(1→		[29]
12	M2	DEAE-Cellulose, HW-55F, Sephacryl S-400	1800.5	Fru, Glc in the ratio of 10:1	→1,6)- Fru*f*-(2→, α-d-Glc*p*-(1→	Hypoglycemia	[30]
13	Abs	Sephadex G-50 column	1400	Fru, Glc	2→6 linked and 2→1 linked*β*-d-Fru*f* residues		[31]
14	ABAB	Cellex D, Sephadex G-150 column	23,000	GlcA, Gal, GalA, Ara, Rha in the ratio of 12:2:3:1:1	→1)-d-Glc*p*A-(4→, →4)-d-Gal*p*A-(1→		[32]
15	ABP90-2	DEAE-Cellulose 52 Sepharose S-100 column	2976	Fru, Glc in the ratio of 16:1	α-d-Glc*p*-(1→, *β*-d-Fru*f*-(2→, →1,6)-*β*-d-Fru*f* -(2→, →1)-*β*-d-Fru*f*-(2→, →2)-*β*-d-Fru*f*-(6→		[21]
16	ABP50-2	DEAE-Cellulose 52 Sepharose S-100 column	5118	Fru, Glc in the ratio of 27:1	α-d-Glc*p*-(1→, *β*-d-Fru*f*-(2→, →1,6)-*β*-d-Fru*f*- (2→, →1)-*β*-d-Fru*f*-(2→, →2)-*β*-d-Fru*f*-(6→		[21]
17	FTN-5	DEAE-Cellulose 52	10,000	Man:Rha:Rib:GlcA:GalA:Glc:Gal:Xyl:Ara:Fuc in the ratio of 1.21:2.13:0.38:0.79:19.38:2.32:2.42:1.00:0.97:0.36			[33]
18	OTN-6	DEAE-Cellulose 52	22,400	Man:Rha:Rib:Glc:Gal:Ara:Xyl in the ratio of 1.27:0.73:0.71:1.84:2.06:0.60: 1.01			[3,34]
19	OTN-5	DEAE-Cellulose 52	30,800	Man:Rha:Rib:GalA:Glc:Gal:Xyl:Ara:Fuc in the ratio of 2.99:4.20:0.48:4.85:5.28:4.89:1.64:1.87:0.43			[3]
20	TTN-5	DEAE-Cellulose 52	97,700	Man:Rha:Rib:GalA:Glc:Gal:Ara:Xyl:Fuc in the ratio of 0.67:3.22:0.33:10.73:1.73:4.53:2.44:1.78:0.36			[3]
21	TTN-6	DEAE-Cellulose 52	46,800	Man:Rha:Rib:GlcA:GalA:Glc:Gal:Ara:Xyl:Fuc in the ratio of 1.53:1.24:0.37:2.96:1.43:8.32:4.00:0.86:1.33:0.29			[3]
22	TTN-7	DEAE-Cellulose 52	57,100	Man:Rha:Rib:GlcA:GalA:Glc:Gal:Ara:Xyl:Fuc in the ratio of 1.96:1.98:0.39:1.27:2.30:6.60:4.71:1.68:1.85:0.30			[34]

Rha: rhamnose, GlcA: glucuronic acid, GalA: galacturonic acid, Glc: glucose, Gal: galactose, Ara: arabinos, Fru: fructose, Rib: ribose, Man: mannose, Xyl: xylose, Fuc: fucose.

## Data Availability

No data were used for the research described in the article.

## Supplementary Materials

The following supporting information can be downloaded at: https://www.mdpi.com/article/10.3390/molecules30122523/s1, PRISMA 2020 abstract checklist, PRISMA 2020 checklist and PRISMA 2020 flow diagram. Reference [93] has been cited in the Supplementary Materials.
