# Supplementary material for: Isolation, Structures, and Bioactivities of Polysaccharides from Achyranthes bidentata: A Review
Source: Molecules. 2025 Jun 9;30(12):2523. doi: 10.3390/molecules30122523 (PMC12196059; doi:10.3390/molecules30122523)
Supplement: Supplementary file 1 [file molecules-30-02523-s001.zip › molecules-3660165-PRISMA_2020_flow_diagram.pdf]

PRISMA 2020 flow diagram for new systematic reviews which included searches of databases, registers and other sources

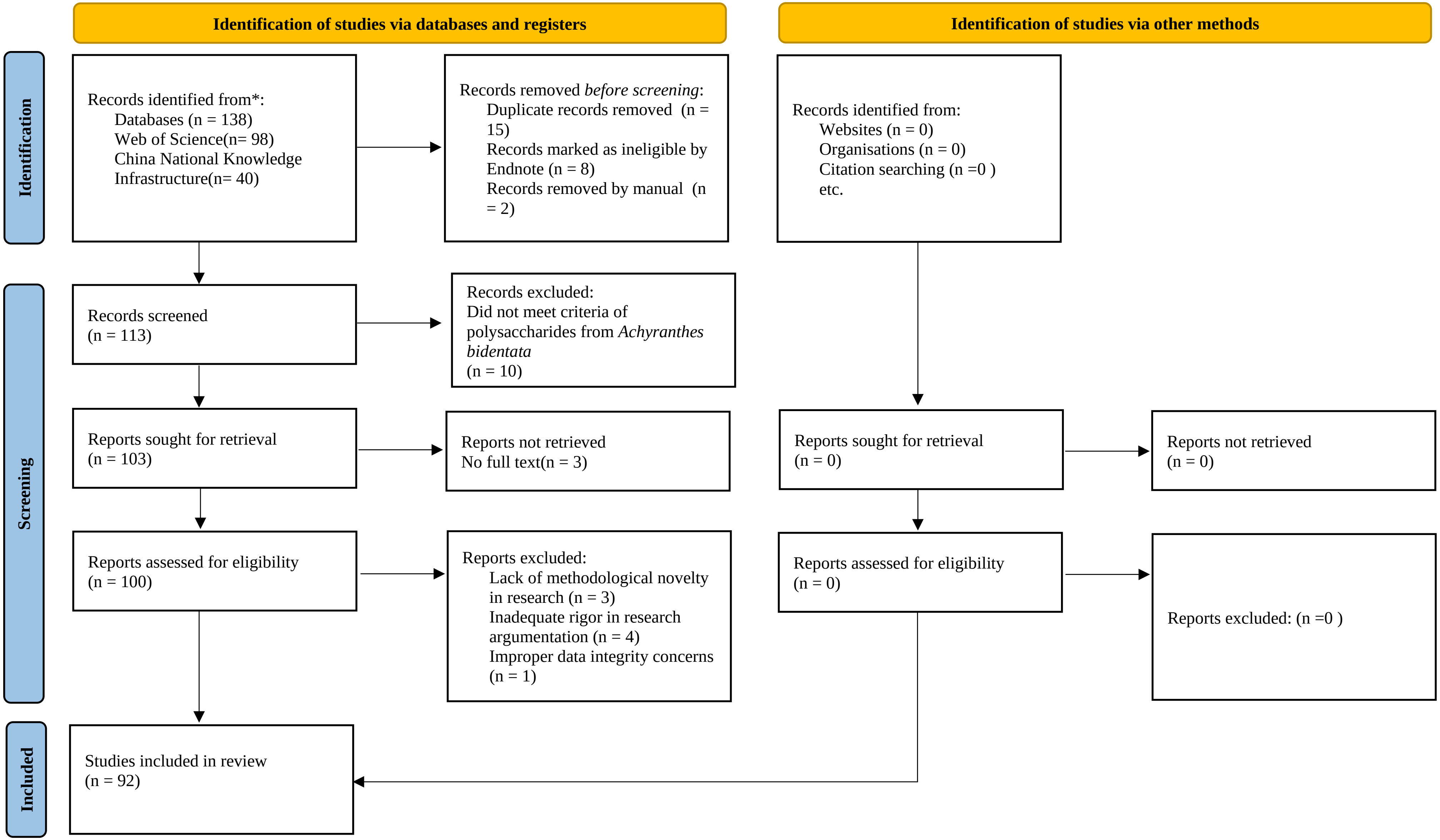

Figure 1 PRISMA- Literature Screening and Inclusion Flowchart

*Consider, if feasible to do so, reporting the number of records identified from each database or register searched (rather than the total number across all databases/registers).
**If automation tools were used, indicate how many records were excluded by a human and how many were excluded by automation tools.

Source: Page MJ, et al. BMJ 2021;372:n71. doi: 10.1136/bmj.n71.

PRISMA 2020 flow diagram for new systematic reviews which included searches of databases, registers and other sources

This work is licensed under CC BY 4.0. To view a copy of this license, visit https://creativecommons.org/licenses/by/4.0/
